# The role of LGR4 in bone metabolism and tumor bone metastasis

**DOI:** 10.3389/fendo.2025.1541601

**Published:** 2025-05-21

**Authors:** Jiawang Huang, Yucheng Jin, Zhigang Yi

**Affiliations:** ^1^ The Second Medical College of Lanzhou University, Lanzhou, China; ^2^ Lanzhou University Second Hospital, Lanzhou, China

**Keywords:** LGR4, bone metabolism, bone metastasis, osteoclasts, osteoblasts

## Abstract

The Leucine-rich repeat-containing G protein-coupled receptor 4 (LGR4) is a member of the G protein-coupled receptor family and plays an important role in bone metabolism and tumor bone metastasis. LGR4 affects bone metabolism by regulating the differentiation and activity of osteoblasts and osteoclasts, and is involved in the balance between bone resorption and bone formation. Deficiency of LGR4 leads to osteoporosis, whereas the up-regulation of LGR4 may help to alleviate the development of traumatic osteoarthritis. Furthermore, in breast cancer and multiple myeloma, LGR4 promotes tumor cell metastasis to bone tissue by activating related signaling pathways. Therefore, LGR4 may be a potential target for the treatment of bone metabolic diseases and tumor bone metastasis.

## Introduction

G Protein-Coupled Receptors (GPCRs), a class of receptor proteins located on cell membranes, are able to sense extracellular signaling molecules (e.g., hormones, neurotransmitters, light, odors, etc.) and convert them into intracellular signals to regulate cellular functions and behaviors (1). It plays a key role in cellular signaling. GPCRs, also known as 7-trans-membrane (7-TM) receptors, are divided into three groups according to their function and structure (2, 3). Group A includes LGR1 recognizes follicle-stimulating hormone. LGR2 recognizes luteinizing hormone. LGR3 recognizes thyroid-stimulating hormone. Group B includes LGR4, LGR5, and LGR6 receptors. It plays a key role in development and have been implicated in a variety of metabolic disorders and cancers (4). Group C includes LGR7 recognizes the receptors in the LGR7 (RXFP1 receptor) and LGR8 (RXFP2 receptor) classes of insulin peptides and relaxin in the LGR8 (RXFP2 receptor) class of insulin peptides (5).

LGR4 is a leucine-rich repeat sequence of G protein-coupled transmembrane receptors, belonging to the superfamily of GPCRs (3). The helical structural domains of all GPCRs share a common sevenfold transmembrane structure, which is also shared by LGR4 (6). The extracellular region consists of the N-terminus and three extracellular loops (ECL1-ECL3), the transmembrane region consists of seven α-helices (TM1-TM7), and the intracellular region consists of three intracellular loops (ICL1-ICL3), an intracellular amphipathic helix (H8), and the C-terminus (3).

*Lgr4* gene was first characterized in 1998 and is located on human chromosome 11 (11 p14.1) (5), encoded by a highly conserved 106827 pb gene, containing 18 exons and 17 introns (6). LGR4 is widely expressed in a variety of tissues, including bone, brain, mammary gland and thymus (7, 8). The main functions involve energy metabolism, bone formation and remodeling, and tumorigenesis. Meanwhile, LGR4 has important effects on development and growth in physiological processes including eye, skin, heart, digestive system, reproductive system, hematopoietic system, etc (9–16). More importantly, the author and other researchers found that LGR4 plays an important role in the pathophysiology of bone metabolic disease and tumor bone metastasis.

## LGR4 in bone metabolic diseases

**Figure 1:**
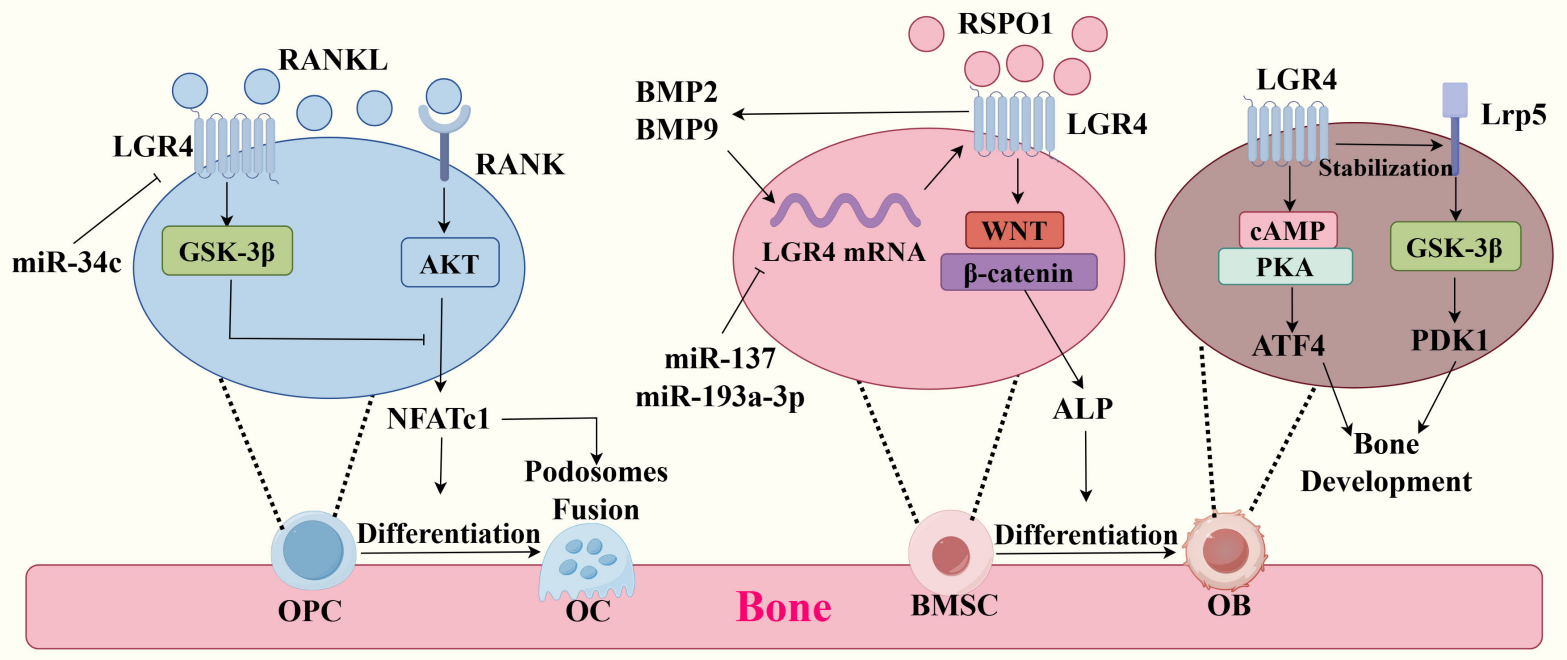
Molecular mechanisms of LGR4 in bone metabolism. (Left) Competitive binding of LGR4 and RANK to RANKL activates the GSK-3β signaling pathway and inhibits the RANKL-RANK signaling pathway, which inhibits the production of the transcription factor NEATC1, thereby inhibiting differentiation of osteoclast precursor cells (OPCs) to osteoclasts (OCs) and podosomes fusion. miR-34c could have been the transcription of *lgr4* mRNA. (Middle) RSPO1 binding to LGR4 activates Wnt/β-catenin, which promotes ALP production and the differentiation of bone marrow mesenchymal stem cells (BMSCs) to osteoblasts(OBs). miR-137 and miR-193a-3p could have been the transcription of *lgr4* mRNA, inhibiting this process. BMP9 and BMP2 could promote the expression of LGR4 and the expression of LGR4 in turn promotes their production. (Right) LGR4 activates the cAMP-PKA signaling pathway by binding to ligands and thus promotes the secretion of ATF4, which is essential for bone formation and development. And LGR4 can Stabilize with Lrp5 and activate the GSK-3β signaling pathway, which promotes the expression of PDK1 and bone development.

Bone formation involves several basic steps: formation of ossification centers, cartilage ossification, endosteal ossification, intramembranous ossification, and bone remodeling. After growth and development, the process of bone remodeling allows the skeleton to adapt to the needs of the organism and maintain its strength and shape. And most bone metabolic diseases are due to disturbances in the bone remodeling process. Bone remodeling includes bone resorption and bone formation (17). The balance and constraint between bone resorption and bone formation helps maintain homeostasis within the bone. when bone resorption is over-activated or bone formation is inhibited, skeletal diseases such as osteoporosis occur. LGR4 plays an essential role in bone resorption and bone formation (**Figure 1**, **Table 1**).

**Table 1:** The role of LGR4 in bone metabolism.

Function	Related Pathways/Molecules	Clinical Relevance	References
Osteoclast Differentiation inhibition	RANKL-RANK- NFATc1, GSK- 3β pathway	Potential anti-bone loss target	(22, 29, 31)
Podosomes Fusion inhibition	RANKL-RANK- NFATc1, GSK- 3β pathway	Relevance to osteoporosis, potential anti-bone loss target	(22, 29, 31, 34–36)
Osteoblast Differentiation	Wnt-β-catenin- ALP, LGR4-ZNRF3	Potential targets for the treatment of osteoporosis	(18, 19, 41)
Bone Homeostasis Maintenance	cAMP-PKA-ATF4 pathway	Core Regulators of Bone Metabolic Diseases	(23)
Glycolysis Regulation	Lrp5-Gsk-3β- PDK1	A new mechanism for metabolic bone disease	(48)
BMP Signal Interaction(Bone protection)	mTORC1/Stat3 pathway, BMP2, BMP9	Synergistic promotion of bone formation	(49, 50)

### Bone resorption

The key to bone resorption lies in the process of resorption and degradation of bone tissue by osteoclasts (OC). OC are derived from monocyte/macrophage lineage in hematopoietic stem cells (18). LGR4 inhibits OC differentiation and bone resorption (19–21). In the past, the studies have shown that knockout of LGR4 results in increased OC activity and bone loss in mice (22–24). Increased postnatal OC activity in LGR4-deficient mice (25). Both systemic and monocyte-specific LGR4 knockout mice exhibited increased OC activity and bone erosion. They were characterized by OC overactivation, increased proliferation, and decreased apoptosis (22). It has been suggested that compressive forces can promote OC differentiation by decreasing LGR4 expression, as LGR4 deficiency increases the number and size of OC (26). Furthermore, MicroRNA-34c promotes OC differentiation by targeting LGR4 (27).

Activation of the RANK signaling pathway is a key component of OC activation. Previously, we thought that RANKL was the only ligand for RANK. Activation of the RANK-RANKL signaling pathway promotes osteoclast differentiation and bioactivity, and clinical treatment of osteoporosis with denosumab, a monoclonal antibody to RANKL (28). RANKL activates the RANK-AKT-NFATc1 cascade signaling response and promotes osteoclast differentiation (29). However, the study has found Mutant RANKL (MT RANKL) binds with high affinity to LGR4 and inhibits RANKL-induced OC formation via an LGR4-dependent pathway (29, 30). LGR4 competes with RANK for binding RANKL and inhibits the RANK signaling pathway during OC differentiation (22, 29). In addition, RANKL binding to LGR4 activates the GSK-3β signalling pathways, thereby inhibiting the expression and activity of NFATc1, a key transcription factor for osteoclastogenesis (20, 22). Recent studies have found that in osteoclast precursor cells, LGR4 binds to RANKL and then enters intracellular endosomes via endocytosis. This process is essential for regulating RANKL signaling (31). It’s worth mentioning that miR-34c promotes osteoclast differentiation through targeting LGR4 (27).

Podosomes are critical to the function of OCs. Podosomes are cellular structures in OCs composed of actin that are involved in functions such as adhesion, matrix degradation, mechanosensing, cell migration, and fusion (32). The structure of podosomes includes sub-structural domains such as cores, loops, and caps, with specific proteins comprising each domain (33). Numerous studies have shown that the RANKL-RANK signaling pathway promotes the formation of podosomes by osteoclasts through integrin αvβ3, NFATc1, CTSK, and other pathways, thereby promoting bone resorption (34–36). As mentioned previously, LGR4 can competitively inhibit the RANKL-RANK signaling pathway, resulting in less NFATc1 production (22, 29). It can be inferred that LGR4 can reduce the production of podosomes through this process, thereby attenuating osteolysis. The specific molecular mechanism remains to be investigated.

### Bone formation

Several studies have indicated a critical role of LGR4 in bone formation and bone mass maintenance (6). A study based on a Chinese family association analysis found a significant correlation between the *lgr4* gene and peak bone mineral density (total hip BMD, lumbar spine BMD) (37). One study even suggests that upregulating LGR4 may help alleviate the development of joint inflammation in traumatic osteoarthritis (38). Delayed bone formation and significantly affected bone remodeling in mouse embryos knocked down for LGR4, with decreased bone formation rate, bone mineral density, reduced mineralization (23). It is now known that the effects of LGR4 on bone formation are primarily mediated through osteoblasts (OB) and bone morphogenetic protein.

OB differentiation is significantly reduced in mice with knockout of the *lgr4* gene (23). Hydrogen peroxide(H_2_O_2_) treatment inhibits *lgr4* mRNA expression in osteoblast MC3T3-E1 cells, thereby affecting osteoblast phenotype (39). Osteoblasts are derived from bone marrow mesenchymal stem cells (BMSCs) (18). LGR4 is expressed in BMSC and OB (40). LGR4 deficiency has been demonstrated to reduce bone mass by inhibiting the differentiation of bone marrow-derived stem cells (BMSCs) into OB and by impeding their migration and apoptosis. LGR4 is highly expressed during embryonic development and in osteoblasts (40). Its significance lies in the fact that LGR4 regulates the cAMP-PKA signalling pathway and the expression of the transcription factor Atf4, which is essential for OB function and bone development (23). Interestingly, the binding of RSPO1 and its receptor LGR4 activated the downstream Wnt/β-catenin signaling pathway and increased the expression of osteogenic genes, such as ALP, under the stimulation of continuous cyclic mechanical stretch (CMS), which promotes the differentiation of BMSCs towards OB (19). Activation of the Wnt/β-catenin signalling pathway by LGR4 is an important mechanism to promote bone formation, but R-spondin ligands need to bind to both LGR4 and ZNRF3/RNF43 to activate Wnt signalling by inducing the removal of ZNRF3/RNF43 from the cell membrane and stabilising the Wnt receptor (18, 41). In addition, microRNAs (miR-137 and miR-193a-3p) were reported to bind to the 3’-UTR of *lgr4* mRNA and block OB differentiation by inhibiting LGR4 transcription (42, 43).

Osteoblasts, even under adequate aerobic conditions, primarily undergo aerobic glycolysis, a phenomenon known as the Warburg effect, so aerobic glycolysis is essential for osteoblasts (44–47). Recent studies have found that LGR4 promotes the expression of pyruvate dehydrogenase kinase 1 (Pdk1), a key enzyme in aerobic glycolysis, and regulates bone remodelling through Lrp5-Gsk3β/β-catenin signalling. Mice specifically deficient in LGR4 showed impaired glycolysis in osteoblasts, resulting in reduced bone mass and strength, and the activated typical Wnt/β-catenin signalling pathway could rescue the glycolytic dysfunction caused by LGR4 deficiency. Mice specifically deficient in LGR4 exhibit impaired osteoblast glycolysis leading to reduced bone mass and bone strength, and activation of the typical Wnt/β-catenin signaling pathway rescues the glycolytic dysfunction caused by LGR4 deficiency (48). The above studies have shown that LGR4 is able to respond to various extracellular signals and activate downstream signalling pathways (cAMP-PKA, Lrp5-Gsk-3β/β-catenin and Wnt/β-catenin) regulating osteogenic differentiation and bone formation.

Some studies have reported that LGR4 is also closely related to bone morphogenetic protein (BMP), especially BMP2 and BMP9 (49, 50). BMP2 can enhance the expression of LGR4 gene through transcriptional regulation, which in turn is required for BMP-induced osteoblast differentiation (49). BMP9 induces LGR4 expression in BMCS through the mTORC1/Stat3 signalling pathway for differentiation to osteoblasts (50). In unison, LGR4 may indirectly affect the role of BMP signalling in tooth development by regulating other signalling pathways (e.g. FGF and Shh) during tooth development (51). Sone showed that specific knockdown of LGR4 in uterine stromal cells significantly reduced BMP2 levels (52). In summary, BMP enhances the expression of LGR4, which in turn is affected by LGR4, and this regulatory relationship may play an important role in the regulation of bone metabolism and bone mass, but its specific molecular mechanism is not clear at present.

Eleven years have passed since the first report that nonsense mutations in the LGR4 gene were strongly associated with low bone mineral density and osteoporotic fractures (25). During this time, evidence has continued to show that LGR4 deletions are highly associated with osteoporosis. LGR4 exhibits corresponding functions in bone resorption and bone formation, for which we can consider it as a potential target for the treatment of osteoporosis. However, there is still a lot of work to be done, including but not limited to constructing humanised mouse models carrying homozygous human LGR4 mutations, identifying new endogenous LGR4 ligands to explore their unique downstream signalling pathways, etc.

### LGR4 in tumor bone metastasis

Bone is a common site of metastasis for many solid tumors, including breast cancer, multiple myeloma (MM), prostate cancer, lung cancer, renal cancer, and thyroid cancer (53). Bone metastases are 65-80% more prevalent in breast and prostate cancers (54). More seriously, about 80% of MM patients have multiple myeloma bone disease as their first symptom (55). Once tumors metastasize to the bone, they are a major cause of morbidity and mortality (56). Bone metastasis can lead to a series of clinical complications such as pathologic fracture, spinal cord and nerve root compression, osteoporosis, hypercalcemia, and bone pain, which have a serious impact on the quality of life of the patients and are closely related to the prognosis of the disease and the survival of the patients (57).

**Figure 2:**
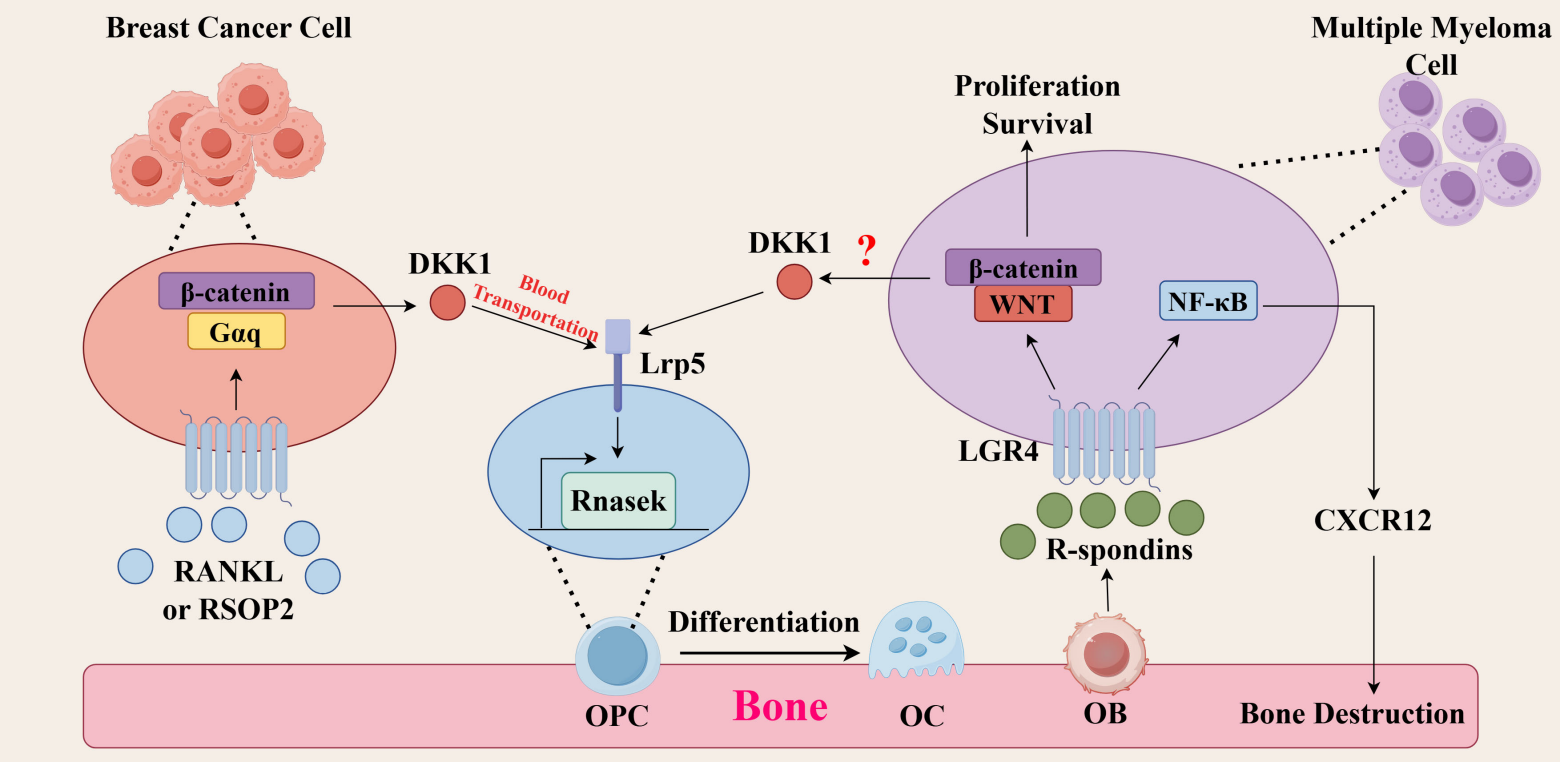
Molecular mechanisms of LGR4 in tumor bone metastasis. (Breast cancer) RSPO2 or RANKL binds to LGR4 on breast cancer cells, activates the Gαq/β-catenin signaling pathway, promotes the secretion of DKK1, which is transported through the bloodstream to the bone matrix, and then binds to LRP5 on osteoclast precursor cells (OPCs), promoting the secretion of the chemokine Rnasek, which promotes bone metastasis of breast cancer. (Multiple myeloma) R-spondins secreted by osteoblasts bind to LGR4 on multiple myeloma cells and activate the Wnt/β-catenin signaling pathway, which promotes the survival and proliferation of multiple myeloma and its bone metastasis. Whether this pathway can generate DKK1 remains to be investigated. Meanwhile, LGR4 can activate the NF-κB signaling pathway and promote the production of CXCR12, leading to osteolytic lesions.

LGR4 is a double-edged sword. How positively he plays in bone homeostasis is how negatively he plays in tumors. LGR4 expression is upregulated in cancers, including breast, prostate, MM, colon, lung adenocarcinomas and high-grade plasmacytoid ovarian cancer, and plays a role in tumor progression, invasion, and metastasis (58–64). This article focuses on its role in tumor invasion and metastasis, especially bone metastasis. However, the current studies on the role of LGR4 in tumor bone metastasis are mainly focused on breast cancer and MM (**Figure 2**, **Table 2**).

**Table 2:** The role of LGR4 in tumor bone metastasis.

Tumor type	Function	Related Pathways/Molecules	Clinical Relevance	References
Various tumors	Tumor Metastasis	——	Metastasis risk marker	(58–63)
Breast, Prostate	Promoting EMT	E-cadherin, Slug, vimentin	Bone metastasis risk markers	(42, 58)
Breast, Multiple myeloma*	DKK1 secretion	Gαq/β-catenin pathway	Key factors in the microenvironmental regulation of bone metastasis	(69, 74)
Multiple myeloma	Osteolytic lesion, Proliferation, Survival	NF-κB, Wnt/β-catenin pathway	Multiple myeloma therapeutic targets	(71, 72)

*The mechanism is not yet clear.

### Breast cancer bone metastasis

Epithelial-mesenchymal transition (EMT) is a cell biological process that refers to the gradual loss of cell-cell connectivity and cell polarity of epithelial cells and the acquisition of mesenchymal cell characteristics, which in turn are transferred to distal organs (65). This process plays a key role in the enhanced migratory and invasive capacity tumors, allowing tumor cells to detach from the primary site, enter the bloodstream or lymphatic system, and form metastases in distant organs (66–68). In breast cancer, whereas one study found that LGR4 correlated with the expression of EMT markers, both the epithelial marker E-cadherin was downregulated and the mesenchymal markers Slug and vimentin were upregulated (58). In prostate cancer, LGR4 similarly promotes EMT (42). Loss of Lgr4 expression was also found to delay breast tumorigenesis, progression and metastasis formation (58).

A positive correlation between LGR4 expression and the occurrence of bone metastasis in breast cancer was found by bioinformatics. Analysis of LGR4 expression in breast cancer monoclonal subline cell lines with different bone metastatic capacities revealed that LGR4 expression was positively correlated with the potential bone metastatic capacity of breast cancer cells, and likely promoted the occurrence of bone metastasis in breast cancer. The molecular mechanism is that R-spondin 2 (RSPO2) and RANKL activate the Gαq/β-catenin signaling pathway by binding to the receptor LGR4, which regulates the expression of DKK1, which binds to LRP5 on osteoclast precursor cells to stimulate the secretion of Rnasek, a chemokine, and promotes bone metastasis of breast cancer (69).

### Multiple myeloma bone disease

Multiple myeloma bone disease (MMBD) is a bone lesion caused by multiple myeloma, which manifests itself primarily as an osteolytic lesion that is similar to bone metastases caused by solid tumors (70). The exact mechanism of action of LGR4 in MMBD is not clear, and its role can only be hypothesized at this time by some of the available evidence. Recent studies have found that LGR4 is highly expressed in multiple myeloma cells but largely unexpressed in plasma cells (71). LGR4 interacts with and regulates the expression of TGF-β1, activates the TGF-β1/Smad signaling pathway and promotes multiple myeloma progression (14). At the same time, aberrantly expressed LGR4 empowers Wnt signaling in multiple myeloma by hijacking osteoblast-derived R-spondins, thereby promoting the development of multiple myeloma (71). Recent studies have shown that MM cells overexpressing LGR4 activate the NF-κB signaling pathway, which promotes the up-regulation of CXCR12 adhesion-related molecule gene expression and adhesion to bone marrow stromal cells, promoting bone destruction (72).

Traditional studies have suggested that the RANK/RANKL signaling pathway plays an irreplaceable role in multiple myeloma bone disease (73). In studies on osteoporosis, we found that LGR4 can bind to RANKL (22, 73). Then it is doubtful whether LGR4 can bind to RANKL and thus promote MMBD.A 20-year-old study found that myeloma cells can secrete the factor DKK1, which affects the function of osteoblasts and osteoclasts, leading to the development of MMBD (74). Interestingly, in studies of breast cancer, LGR4 has been found to promote DKK1 secretion, which promotes breast cancer bone metastasis (69). Whether this effect could also play a role in MMBD deserves to be investigated.

In conclusion, LGR4 plays an important role in tumor bone metastasis and is a potential target worthy of further in-depth study. The regulation of LGR4 may become a new strategy for the prevention and treatment of tumor bone metastasis. However, the regulatory mechanism of LGR4 in tumor bone metastasis is still incomplete, and further in-depth studies on the signaling pathways and regulatory networks downstream of LGR4 are needed.

## Potential clinical value

LGR4 has great potential in the treatment of bone metabolic disorders. Bone metabolic disorders include osteoporosis, osteomalacia, etc. LGR4 is involved in the regulation of bone metabolic homeostasis, and the modulation of its signaling pathway provides new ideas for the treatment of bone metabolic diseases. In the genetic testing given to patients, rare nonsense mutation (c.376C>T) in the *lgr4* gene is significantly associated with low bone mineral density and osteoporotic fracture risk (25). In a case report of osteoporosis in a woman, Poonam et al. found that a mutation (c.1403A>C) was identified in the *lgr4* gene, resulting in a change in residue 468 of the protein from tyrosine to serine (75). Whether the normal expression of *lgr4* can be restored by gene editing technology to treat osteoporosis is worth studying. In terms of drug development, some researchers have replaced the histidine at positions 223 and 224 of RANKL with phenylalanine and tyrosine respectively, so that it cannot bind to RANK but only to LGR4 (76). This modified RANKL has played a very good role in the treatment of osteoporosis in mice, and its clinical application is very worthy of research. Injecting mice with soluble LGR4-ECD, a free protein that binds to ligands but does not activate signaling pathways, can treat osteoporosis (22). This may be related to the fact that LGR4-ECD binds RANKL and inhibits osteoclast differentiation. The clinical applications of LGR4-ECD are also very promising.

In the treatment of tumor bone metastasis, LGR4 also has important application value. Tumor bone metastasis is a common complication in many cancer patients, which seriously affects the quality of life and prognosis of patients. LGR4 plays a key role in promoting the metastasis of tumor cells to bone tissues, and the blockage of its signaling pathway may become an effective means for the treatment of tumor bone metastasis. Soluble LGR4-ECD protein, containing the binding domains of RSPO2 and RANKL, significantly inhibits bone metastasis (69). This soluble LGR4-ECD for clinical treatment of tumor bone metastases is very promising for application. In bone metabolism studies, we found that multiple microRNAs (e.g. miR-137) reduced LGR4 expression (27, 42, 43). Whether these microRNAs can be applied in tumors to reduce the expression of LGR4 and decrease the bone metastasis of tumors is also worth investigating. By developing monoclonal antibodies and small molecule inhibitors against LGR4, we can inhibit the growth and metastasis of tumor cells in bone tissues, alleviate patients’ pain and skeletal complications, and improve patients’ survival rate and quality of life.

## Conclusion and outlook

LGR4 plays a dual role in bone metabolism and tumor bone metastasis, both in maintaining bone metabolic homeostasis and promoting tumor cell metastasis to bone tissue. Although a large number of studies have confirmed the importance of LGR4 in these two processes, its specific molecular regulation mechanism still needs to be further investigated. In the future, by constructing mouse models carrying humanized LGR4 mutations and discovering new endogenous LGR4 ligands, the unique signaling pathway of LGR4 can be further elucidated, which will lay the foundation for the application of LGR4 in the treatment of bone metabolic diseases and tumor bone metastasis. It is also important to translate basic research on LGR4 into clinical applications, and the development of drugs and therapeutic technologies targeting LGR4 is both promising and important.
